# Submaximal measures of fitness and left ventricular size: associations and utility in differentiating physiological and pathological enlargement

**DOI:** 10.1093/ehjimp/qyag081

**Published:** 2026-04-27

**Authors:** Julie Stendahl Spets, Victoria Elisa Joakimsdatter Sørgjerd, Bjarne Martens Nes, Knut Asbjørn Rise Langlo, Inger-Lise Aamot Aksetøy, Kari Margrethe Lundgren, Ulrik Wisløff, Håvard Dalen, Jon Magne Letnes

**Affiliations:** Department of Circulation and Medical Imaging, Norwegian University of Science and Technology, NTNU, Postbox 8905, Trondheim 7491, Norway; Department of Circulation and Medical Imaging, Norwegian University of Science and Technology, NTNU, Postbox 8905, Trondheim 7491, Norway; Department of Circulation and Medical Imaging, Norwegian University of Science and Technology, NTNU, Postbox 8905, Trondheim 7491, Norway; Department of Cardiology and Cardiothoracic Surgery, St. Olavs University Hospital, Trondheim, Norway; Department of Nephrology, Clinic of Medicine, St. Olavs Hospital, Trondheim University Hospital, Trondheim, Norway; Department of Clinical and Molecular Medicine, Norwegian University of Science and Technology, Trondheim, Norway; Department of Circulation and Medical Imaging, Norwegian University of Science and Technology, NTNU, Postbox 8905, Trondheim 7491, Norway; Clinic of Rehabilitation, St. Olavs University Hospital, Trondheim, Norway; National Advisory Unit on Exercise Training as Medicine for Cardiopulmonary Conditions, St. Olav’s Hospital, Trondheim, Norway; Department of Circulation and Medical Imaging, Norwegian University of Science and Technology, NTNU, Postbox 8905, Trondheim 7491, Norway; Department of Circulation and Medical Imaging, Norwegian University of Science and Technology, NTNU, Postbox 8905, Trondheim 7491, Norway; Department of Circulation and Medical Imaging, Norwegian University of Science and Technology, NTNU, Postbox 8905, Trondheim 7491, Norway; Department of Cardiology and Cardiothoracic Surgery, St. Olavs University Hospital, Trondheim, Norway; Department of Medicine, Levanger Hospital, Nord-Trøndelag Hospital Trust, Levanger, Norway; Department of Circulation and Medical Imaging, Norwegian University of Science and Technology, NTNU, Postbox 8905, Trondheim 7491, Norway; Department of Cardiology and Cardiothoracic Surgery, St. Olavs University Hospital, Trondheim, Norway

**Keywords:** heart volumes, endurance training, scaling, anaerobic threshold, cardiac remodelling, body composition

## Abstract

**Aims:**

Peak oxygen uptake (VO_2peak_) has been shown to better differentiate between physiological and pathological left ventricular (LV) remodelling compared to indexing to body surface area (BSA). This study aimed to investigate the relationship between LV end-diastolic volume (LVEDV) and submaximal measures of cardiorespiratory fitness (oxygen uptake at the ventilatory threshold; VO_2VT_, and at the cardiorespiratory optimal point; VO_2COP_), and to evaluate whether these measures could serve as alternatives for indexing to VO_2peak_.

**Methods and results:**

Transthoracic echocardiography and cardiopulmonary exercise testing (CPET) were performed in 1067 health-survey participants free from cardiovascular disease, 39 healthy recreational athletes, and 53 patients with heart failure. Pearson’s correlation and linear regression were performed to evaluate associations between LVEDV and VO_2VT_, VO_2COP_, VO_2peak_, and BSA. Indexing methods were compared for their ability to classify LV size. The VO_2VT_ (*R*^2^ = 0.44) and VO_2COP_ (*R*^2^ = 0.41) showed stronger correlations with LVEDV and explained a greater proportion of its variance than BSA (*R*^2^ = 32%, both *P* < 0.01), although weaker than VO_2peak_ (*R*^2^ = 0.52, *P* < 0.01). Indexing LVEDV to VO_2VT_ and VO_2COP_ significantly increased the proportion of patients with heart failure classified with enlarged ventricles compared to BSA, and reduced misclassification among athletes. The submaximal measures performed similarly to VO_2peak_ in this classification.

**Conclusion:**

Submaximal measures of fitness (VO_2VT_ and VO_2COP_) showed a similar ability to distinguish between pathological and physiological LV enlargement as maximal measures (VO_2peak_) and better than BSA. Submaximal measures may offer practical fallback alternatives when maximal exercise testing is not feasible.

## Graphical Abstract

**Graphical Abstract:**
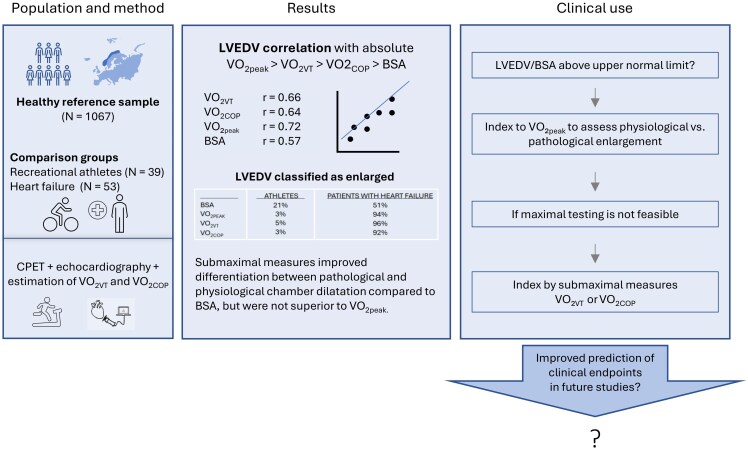

## Introduction

Cardiovascular structures adapt to fulfil metabolic requirements. The association between body size and left ventricular (LV) volume, where LV size increases with body size, is indicative of this relationship.^1^ The size of the LV is expected to scale linearly with metabolic demand as a given oxygen consumption (i.e. energy expenditure) is proportional to cardiac output (CO)—which again is dependent on stroke volume. In line with this, LV enlargement can occur as an adaptive response to repeated exercise stimuli, where increases in LV end-diastolic volumes (LVEDV) facilitate greater stroke volumes, thereby enhancing CO and oxygen uptake.^2–4^ Conversely, LV enlargement may arise as a pathological adaptation to impaired cardiac function, commonly observed in heart failure (HF).^2^ Echocardiographic assessment of LV size is conducted daily in clinical practice. Scaling to body surface area (BSA) is a recommended approach to adjust for differences in body size,^5,6^ although this approach is limited in its ability to distinguish pathological and physiological LV enlargement.^1,7^ In obese individuals, overcorrection for body size may compromise the ability to detect pathology, as differences in body composition are not considered.^1,7^ Conversely, physiological remodelling from exercise can be misinterpreted as pathology.^3,8,9^

Assessing LVEDV in relation to absolute peak oxygen uptake (VO_2peak_) has been shown to improve differentiation between pathological LV enlargement and physiological adaptations.^10^ Being the gold-standard measure of cardiorespiratory fitness, VO_2peak_ can be defined as the highest oxygen uptake achieved during a cardiopulmonary exercise test (CPET).^11^ It is a widely recognized indicator of cardiovascular health,^12^ which directly reflects maximal metabolic demand. However, as there is conflicting evidence as to whether the LV stroke volume progressively increases or plateaus during increasing exercise intensity, it is plausible that submaximal fitness measures may better correlate with LV size.^13^

The first ventilatory threshold (VT) has been shown to correlate well with VO_2peak_,^14^ and is a submaximal measure of cardiorespiratory fitness, representing the oxygen uptake (VO_2_) value corresponding to the onset of arterial blood lactate accumulation.^15^ It can be estimated through interpreting gas exchange data by identifying ventilatory patterns that typically emerge when transitioning to anaerobic metabolism.^15,16^ As estimation of the VT does not require maximal effort, so it may serve as a suitable alternative to VO_2peak_ for patient groups where maximal testing is considered unfavourable or not achievable.^17–20^ Several overlapping terms for first VT (e.g. gas exchange threshold and estimated lactate threshold) exist, for simplicity it will be referred to as VT.^15^ A proposed alternative, submaximal marker for assessing cardiorespiratory fitness is the cardiorespiratory optimal point (COP), defined as the lowest value of the ventilatory equivalent of oxygen (VE/VO_2_) at any given minute during CPET. Typically occurring at 30–50% of VO_2peak_, the COP is considered to reflect the point of most efficient interaction between the respiratory and cardiovascular system.^21^

The objective of this study was to investigate the relationship between LVEDV and two submaximal measures of cardiorespiratory fitness; VO_2_ at VT (VO_2VT_) and VO_2_ at COP (VO_2COP_), and compare this to associations between LVEDV and VO_2peak_ and BSA, respectively. We hypothesized that VO_2VT_ and VO_2COP_ exhibits a similar relationship to LVEDV as VO_2peak_. Secondly, we wanted to explore whether indexing using VO_2VT_ and VO_2COP_ could demonstrate a discriminatory ability comparable to VO_2peak_, in distinguishing a pathologically enlarged LV in patients with HF from remodelling caused by physiological adaptations in recreational endurance athletes.

## Methods

All participants provided written informed consent before inclusion. This study received approval from the Regional Ethical Committee (REK) in Central Norway (REK IDs 2024/82464 and 2016/1597).

### Healthy reference sample

The Trøndelag Health Study (HUNT), formerly known as the Nord-Trøndelag Health Study, is a cohort study collecting health data from the adult population of Trøndelag County, Norway. Since its initiation in 1984, four major waves of data collection have been completed. The fourth wave of HUNT (HUNT4) collected data from 56 042 adults in Nord-Trøndelag between 2017 and 2019.^22^ A sample of participants from the HUNT4 Fitness Study (*n* = 2448), a substudy of HUNT4, serves as a sample of healthy adults for investigating the relationship between submaximal fitness measures and LVEDV in the healthy state (i.e. healthy reference sample). In the HUNT4 baseline examination, all participants completed self-reported questionnaires, underwent clinical examinations and provided blood samples for biochemical analyses. Weight was measured with participants wearing light clothing and no shoes. Height was recorded to the nearest centimetre. Investigations specific to the HUNT 4 Fitness Study included echocardiography and CPET.^10,22^ Inclusion required a medical history free of angina pectoris, myocardial infarction, HF, stroke, atrial fibrillation, chronic obstructive pulmonary disease, use of antihypertensives and diabetes. Additionally, those with an LV ejection fraction (EF) below 50% and a maximal respiratory exchange ratio (RER) of less than 1.0 during CPET were excluded. Participants without available raw data for VO_2VT_ estimation, or with data of insufficient quality for reliable VO_2VT_ estimation, were excluded. This resulted in 1067 participants being retained in this specific sample. Details about the inclusion and exclusion criteria for the HUNT4 Fitness Study have previously been published.^10^

### Athletes

HUNT4 Fitness Study participants who self-reported current or previous involvement in organized endurance sports competitions (e.g. running, cross-country skiing, cycling, and swimming) were excluded from the healthy reference sample and retained in a separate dataset (i.e. ‘Athletes’, *n* = 39). Information on years of systematic endurance training practice and weekly hours of exercise was also gathered by self-reported questionnaires.

### Heart failure patients

**Figure 1:**
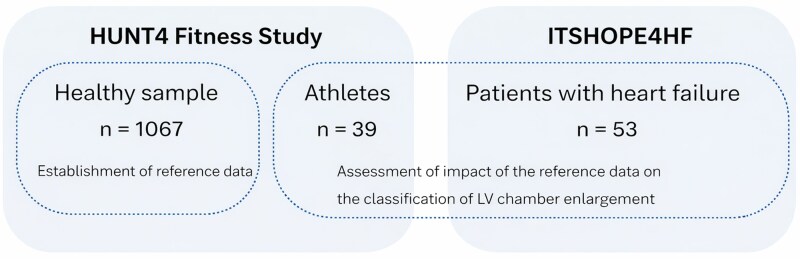
Cohort overview for the present study. HUNT = Trøndelag Health Study; ITISHOPE4HF = Implementation of Telerehabilitation In Support of Home-based Physical Exercise for Heart Failure; LV = left ventricle. *Individuals self-reporting active participation in endurance sports competitions were classified as athletes.

We included a sample of patients ≥18 years with validated HF, participating in ITSHOPE4HF (*n* = 53), a randomized clinical trial reporting on telerehabilitation in HF. Further details regarding inclusion and exclusion have been published.^23^ Data collection at baseline in ITSHOPE4HF involved questionnaires, blood sampling for biochemical analyses, CPET and echocardiography^23^ (*Figure 1*).

### Echocardiography

Echocardiographic data were collected using a comprehensive transthoracic echocardiographic protocol, conforming to current recommendations.^5^ A Vivid E95 scanner (GE ultrasound) with a 4Vc-D matrix transducer was utilized for 2D and 3D imaging.^23,24^ Participants were examined in the left lateral decubitus position, and all echocardiographic recordings included a minimum of three cardiac cycles captured during quiet respiration or breath-hold. The sector depth and width were optimized to focus on the LV while reducing potential errors in delineating the endocardial border. Simpson’s method was utilized for the calculation of LV volumes and EF from 2D recordings of two- and four-chamber views. Agreement of LVEDV measurements was evaluated using Bland-Altman analysis in 37 randomly selected participants with repeated echocardiographic examinations by different operators analysed blindly by the same reader.

Echocardiographic analyses for the HUNT population were performed by two experienced sonographers, while assessments in the group with HF were conducted by a cardiologist. All personnel were connected to the echocardiographic laboratory at St. Olavs Hospital, Trondheim, Norway, which is accredited by the European Association of Cardiovascular Imaging (EACVI).

### Cardiopulmonary exercise testing

Cardiopulmonary exercise testing for the HUNT sample was conducted on a calibrated treadmill, where participants either walked or ran based on fitness and personal preference. Continuous gas analysis was performed throughout the test, using the Metalyzer (Cortex Biophysik GmbH, Leipzig, Germany) portable mixing chamber system, with Metasoft Studio software. The protocol included a 10-minute warm-up. The initial two steps had three- and one-and-a-half-minute duration at a constant load followed by incremental increases in inclination or speed every minute until voluntary exhaustion. Additional details on CPET procedures have been published previously.^25^ For patients with HF, a constant, individualized walking speed was maintained, with the incline increasing every two minutes until exhaustion. The Vyntus CPX system (Erich Jaeger GmbH, Hoechberg, Germany) was used for testing patients with HF.^23^ The Vyntus CPX and the Metalyzer systems used in this study are both part of the same institutional core facility and have been validated against each other to ensure consistency in measurements.

For all groups, test protocols were designed to achieve exhaustion within approximately 10 min. The VO_2peak_ was defined as the highest 30-s average oxygen uptake during the test. The RER was defined as the ratio of exhaled carbon dioxide (L) to oxygen uptake (L) per time unit.

### Estimation of the ventilatory threshold and the cardiorespiratory optimal point

Outputs of 10-s averages of ventilatory variables were obtained directly from CPET and were used to detect the submaximal measures for the HUNT4 participants and patients with HF. An application designed specifically for the detection of exercise thresholds using gas exchange data was employed to find VO_2VT_.^15^ The application allowed for combining different VO_2VT_ determination methods, including the V-slope method, which relies on identifying changes in the VCO_2_–VO_2_ relationship curve.^16^ In addition, the ventilatory equivalent method, along with visual identification of the breakpoint in the VE vs. VO_2_ curve were utilized for estimating VO_2VT_. The integration of several methods has proved to enhance precision in VO_2VT_ detection.^15,16^ The VO_2VT_ value was finally determined by averaging the three measurements, recorded at 10-s intervals, most closely aligned with the estimate from the application. The level of confidence in the VO_2VT_ estimates was graded on an ordinal scale from 1 to 5 based on the clarity and consistency of the gas exchange data, with 1 representing very high certainty, and 5 indicating very low certainty. Participants with VO_2VT_ estimates graded as 5 (*n* = 27 or 11 for HUNT, *n* = 4 for patients with HF *n* = 1 for athletes) were excluded from the statistical analyses, and not counted towards the total reported sample size. The COP was calculated as the lowest 60 s rolling average of VE/VO_2_ during the test. The corresponding VO_2_ value at this point (VO_2COP_) was acquired and utilized in further analysis. The analyses of VO_2VT_ and VO_2COP_ values were done manually by two trained medical students (J.S.S., V.E.J.S.). Bland-Altman analyses were performed to assess interobserver agreement of the manually plotted VO_2VT_ values.

### Statistical analysis

Descriptive data are expressed using either means and standard deviations (SD) or counts and percentages. The associations between LVEDV and VO_2VT_, VO_2COP_, VO_2peak_, and BSA were explored using Pearson’s correlations, reported for the entire population, as well as separately for three distinct age groups (<40 years, 40–60 years, >60 years) and sex. Correlation and univariate regression were also performed separately for the subgroup with the highest confidence in the VO_2VT_ estimates (rated as certainty 1 and 2), as well as for participants reaching a RER between 1 and 1.05 during CPET. Pearsons’s correlation coefficients were compared using Dunn and Clark’s *Z*-test.^26^ To determine the contribution of VO_2VT_, VO_2COP_, VO_2peak_, and BSA to the variance in LVEDV, linear regression analyses were performed with age and sex as covariates.

Classification of normal vs. enlarged LVEDV for athletes and patients with HF was compared across indexing methods, including VO_2VT_, VO_2COP_, VO_2peak_, and BSA, using the upper limits of normal (mean + 2SD) as the classification threshold. Reference values were established based on indexed values retrieved from the healthy reference sample. The chi-squared test was used to assess whether the indexing methods significantly differed from each other. The area under the curve (AUC) statistic was calculated to assess the ability of LVEDV indexed to VO_2VT_, VO_2COP_, VO_2peak_, and BSA, respectively, to differentiate between patients with HF and athletes. This was done using the pROC package in R (www.rproject.org). Other statistical analyses were conducted using StataNow 18.5 MP (StataCorp, TX, USA).

## Results

The 1067 participants from the healthy reference sample comprised 56.7% women with a mean age of 58 (12) years. The mean relative VO_2peak_ was 37.6 (8.7) mL/kg/min, with a VO_2VT_ at 66% (9%) and VO_2COP_ at 63% (10%) of VO_2peak_. The percentage of VO_2VT_ relative to VO_2peak_ was 64% among individuals <40 years and 67% among those >60 years. Among athletes, the female representation was 25.6%, with a mean age of 51 (11) years, and they self-reported performing 27 (15) years of systematic endurance training with an average of 6 (3) hours per week. The patients with HF included 17.0% women with a mean age of 68 (12) years. At least 83% of the HF population received treatment with β-blockers and renin-angiotensin-aldosterone system inhibitors.^22^ Additional demographic characteristics and relevant CPET and echocardiographic data are summarized in *Table 1*.

**Table 1:** General characteristics

Characteristic	Healthy sample, *n* = 1067	Athletes, *n* = 39	Patients with heart failure, *n* = 53
Age	58 (12)	51 (11)	68 (12)
Women	605 (56.7%)	10 (25.6%)	9 (17.0%)
Body mass (kg)	76.1 (13.5)	77.5 (11.6)	85.8 (19.5)
BSA	1.89 (0.20)	1.95 (0.18)	2.02 (0.21)
BMI (kg/m^2^)	25.7 (3.4)	24.4 (2.6)	27.7 (4.8)
Fat-free mass (kg)	54.7 (11.1)	62.7 (11.1)	NA
Systolic BP (mmHg)	128 (18)	122 (14)	119 (20)
Diastolic BP (mmHg)	75 (9)	74 (9)	71 (12)
Resting heart rate (bpm)	68 (11)	59 (11)	69 (11)
Current smoker	38 (3.5%)	0 (0%)	7 (13.2%)
LVEDV biplane 2D (mL)	109 (30)	143 (34)	175 (66)
LVEDV 3D (mL)	116 (29)	142 (26)	NA
LV EF biplane 2D (%)	60.5 (4.3)	60.1 (5.2)	34.7 (10.8)
Cardiopulmonary exercise testing			
Peak oxygen uptake (L/min)	2.88 (0.84)	3.98 (0.86)	1.53 (0.52)
Peak oxygen uptake (mL/kg/min)	37.6 (8.7)	50.9 (7.6)	17.7 (4.6)
Respiratory exchange ratio	1.12 (0.05)	1.11 (0.04)	1.03 (0.09)
Peak heart rate (bpm)	174 (14)	177 (11)	124 (20)
Peak ventilation (L/min)	101 (29)	127 (27)	60 (17)
VO_2VT_ (L/min)	1.88 (0.55)	2.48 (0.62)	1.06 (0.33)
VO_2VT_/VO_2_peak (%)	66 (9)	63 (10)	70 (8)
VCO_2VT_ (L/min)	1.69 (0.53)	2.22 (0.58)	0.85 (0.28)
VT heart rate (bpm)	129 (31)	131 (20)	95 (17)
VT breathing frequency	29 (5)	30 (6)	25 (9)
VT ventilation (L/min)	54 (16)	67 (18)	35 (13)
COP	25.9 (3.0)	24.4 (2.2)	30.4 (5.2)
VO_2COP_ (L/min)	1.78 (0.53)	2.34 (0.68)	1.05 (0.31)
VO_2COP_/VO_2peak_ (%)	63 (10)	61 (13)	71 (12)

Numbers are mean (SD); *n* (%).

BMI, body mass index; BSA, body surface area; BP, blood pressure; COP, cardiorespiratory optimal point (VE/VO_2_); EF, ejection fraction; LV, left ventricle; LVEDV, left ventricle end-diastolic volume; VT, ventilatory threshold; VO_2VT_, oxygen uptake at the ventilatory threshold; VO_2peak_, peak oxygen uptake; VO_2COP_; oxygen uptake at the cardiorespiratory optimal point.

### Submaximal measures of fitness and LVEDV

**Figure 2:**
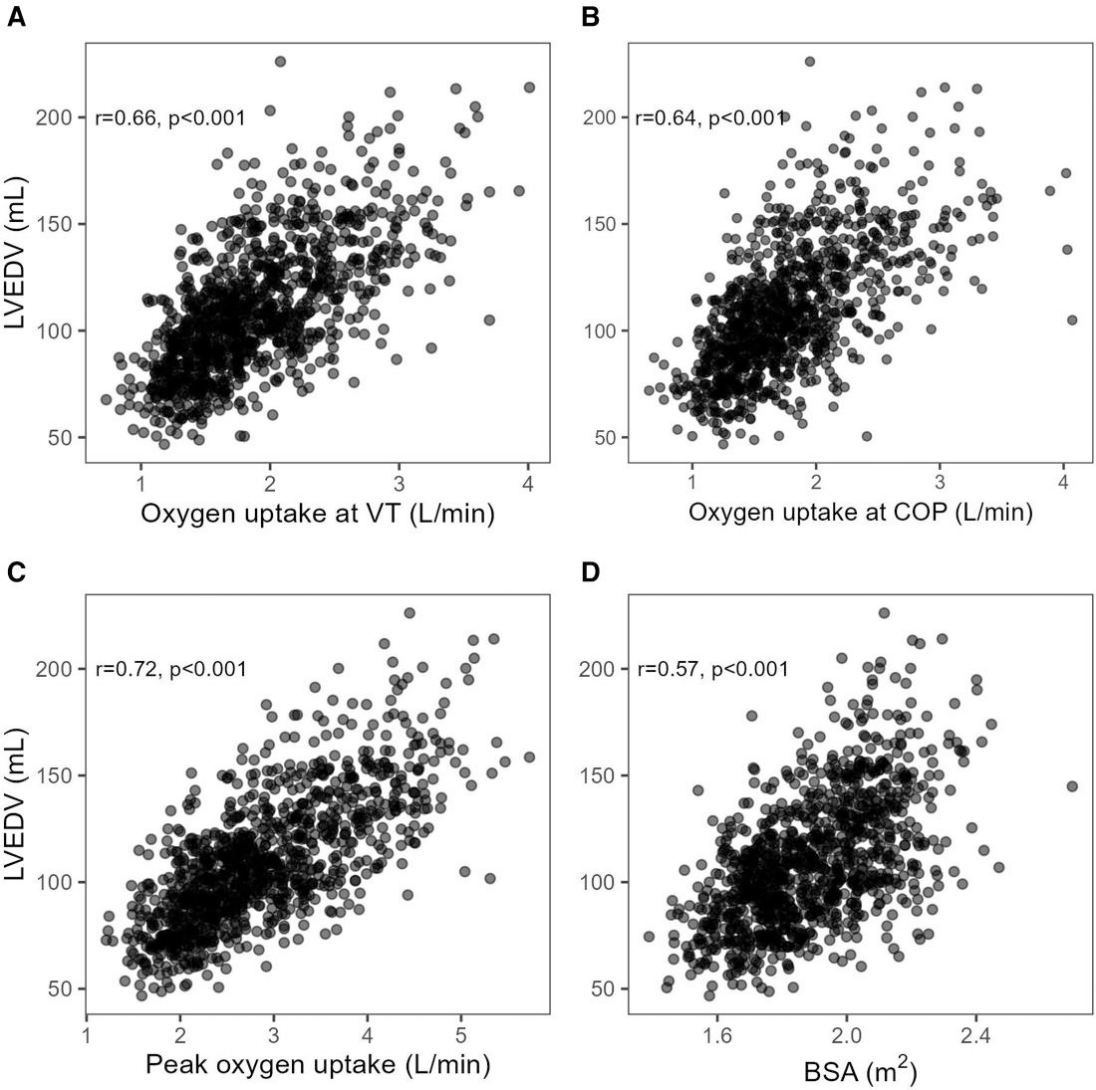
Scatter plots and Pearson’s correlation coefficients for left ventricular end-diastolic volume (LVEDV) vs. (*A*) oxygen uptake at ventilatory threshold (VO_2VT_), (*B*) oxygen uptake at cardiorespiratory optimal point (VO_2COP_) (*C*) absolute peak oxygen uptake (VO_2peak_), and (*D*) body surface area (BSA).

In univariate models, VO_2VT_ and VO_2COP_ explained 44% (*R*^2^ = 0.44, *P* < 0.001) and 41% (*R*^2^ = 0.41, *P* < 0.001), respectively, of the variance in LVEDV, whereas BSA and absolute VO_2peak_ accounted for 32% (*R*^2^ = 0.32, *P* < 0.001) and 52% (*R*^2^ = 0.52, *P* < 0.001) of the variance. When comparing the univariate Pearson’s correlation coefficients, those for VO_2VT_ and VO_2COP_, respectively, and LVEDV were significantly higher than for BSA and LVEDV (both *P* < 0.01, *Table 2*, *Figure 2*). However, the correlation coefficient for absolute VO_2peak_ and LVEDV was significantly higher than those for VO_2VT_ and VO_2COP_ vs. LVEDV (both *P* < 0.01). The correlation coefficient for VO_2VT_ vs. LVEDV was not significantly different from that for VO_2COP_ vs. LVEDV (*P* = 0.44). In the subgroup with the most reliable VT estimates (certainty 1 and 2, *n* = 283), the correlation coefficient increased to 0.74 (*R*^2^ = 0.54) for LVEDV and VO_2VT_, and to 0.78 (*R*^2^ = 0.60) for LVEDV and VO_2peak_.

**Table 2:** Pearson correlation coefficients for LVEDV towards VO_2peak_, VO_2VT_, VO_2COP_, and BSA (*n* = 1067) in the healthy reference sample

Variable	All	Men	Women	<40 yr	40–60 yr	>60 yr
VO_2VT_	0.66*	0.47	0.52	0.56	0.65	0.59
VO_2COP_	0.64*	0.48	0.51	0.55	0.63	0.57
VO_2peak_	0.72^*,†^	0.54	0.61	0.70	0.72	0.65
BSA	0.57	0.30	0.32	0.61	0.53	0.59

BSA, body surface area (m^2^); VO_2VT_, oxygen uptake at the ventilatory threshold (L/min); VO_2COP_, oxygen uptake at the cardiorespiratory optimal point (L/min); VO_2peak_, peak oxygen uptake (L/min); yr, years.

^*^Significantly different from BSA at *P* < 0.05

^†^Significantly different from VO_2VT_ and VO_2COP_ at *P* < 0.05

No significant differences in the Pearson correlations coefficient for VO_2VT,_ VO_2COP,_ VO_2peak_, or BSA between men and women.

Among participants with RER < 1.05, indicating submaximal test effort, the correlation between LVEDV and VO_2peak_ remained consistent (*r* = 0.72), while submaximal measures showed weaker associations with LVEDV (VO_2VT_  *r* = 0.62, VO_2COP_  *r* = 0.59).

### Impact of age and sex

The relationship between LVEDV and VO_2VT_ was examined through multiple linear regression, revealing an influence of both age and sex (*P* < 0.001). When including age and sex in the regression model, the explained variance in LVEDV increased from 44% to 48%. Similarly, age and sex played a significant role in the relationship between VO_2COP_ and LVEDV (*P* < 0.001), with the multivariate model explaining 49% of the variance compared to 41% in the univariate model. When BSA was considered, including age and sex in the model accounted for 45% of the variance in LVEDV, with both variables significantly improving *R*^2^ from 32% in the univariate model (*P* < 0.001). For absolute VO_2peak_, age had no impact on *R*^2^ (*P* = 0.24), while sex made a significant although small contribution (*P* = 0.001, adjusted *R*^2^ = 0.53 compared to 0.52**)**.

Linear regression analyses assessing the relationship between LVEDV/VO_2VT_ and age showed that age explained 1.7% of the variance in LVEDV/VO_2VT_ (*R*^2^ = 0.017, *P* < 0.001), while sex accounted for 1.3% of the variance (*R*^2^ = 0.013, *P* < 0.001). For VO_2COP_, sex had no substantial influence on LVEDV/VO_2COP_ (*P* = 0. 22) and age explained 0.9% (*R*^2^ = 0.009, *P* = 0.001). In comparison, age and sex accounted for 12.2% and 11.1% of the variance in LVEDV/BSA, respectively (both *P* < 0.001). Analyses on LVEDV/VO_2peak_ revealed a 6.5% explanation in variance by age, while sex accounted for 4.5% of the LVEDV/VO_2peak_ variance.

### Classification of normalcy in patients with HF and athletes

Normal ranges for the indexed LVEDV values, including age- and sex-specific data, are presented in *Table 3*.

**Table 3:** Mean and lower (mean − 2SD) and upper (mean + 2SD) limits of normalcy for LVEDV indexed by VO_2VT_, VO_2COP_, VO_2peak_, and BSA in the healthy reference sample

	Women	Men	Overall
LVEDV/VO_2VT_			60.1 (32.2–87.9)
<40 yr	62.8 (32.0–93.5)	54.6 (31.8–77.4)	
40–60 yr	61.3 (37.1–85.6)	55.0 (28.8–81.1)	
>60 yr	61.3 (32.6–90.1)	62.4 (32.4–92.4)	
LVEDV/VO_2COP_			63.5 (33.6–93.4)
<40 yr	65.1 (36.1–94.0)	60.6 (32.2–89.0)	
40–60 yr	64.1 (37.7–90.5)	59.8 (31.7–88.0)	
>60 yr	63.7 (32.2–95.1)	66.4 (33.6–99.2)	
LVEDV/VO_2peak_			39.1 (22.2–56.0)
<40 yr	39.3 (25.2–53.4)	33.6 (22.7–44.4)	
40–60 yr	39.9 (25.6–54.2)	34.5 (21.8–47.2)	
>60 yr	41.7 (23.3–60.1)	40.3 (21.6–59.0)	
LVEDV/BSA			57.7 (31.1–84.2)
<40 yr	64.1 (38.9–89.4)	70.4 (43.6–97.2)	
40–60 yr	57.2 (36.0–78.3)	65.0 (37.6–92.4)	
>60 yr	48.6 (28.9–68.4)	59.2 (34.2–84.2)	

Values are mean (lower limit of normal to upper limit of normal).

BSA, body surface area (m^2^); LVEDV, left ventricular end-diastolic volume; VO_2VT_, oxygen uptake at the ventilatory threshold (L/min); VO_2COP_, oxygen uptake at the cardiorespiratory oxygen uptake (L/min); VO_2peak_, peak oxygen uptake (L/min); yr, years.

When indexing LVEDV to VO_2VT_ and VO_2COP_, the proportion classified as pathologically enlarged among the patients with HF significantly increased, compared to using BSA (*P* < 0.001, *Table 4*). Indexed to VO_2VT_, 51 (96%) of the LVs of the patients with HF were classified as enlarged, whereas 49 (92%) of the population exceeded upper limits when indexed to VO_2COP_. Using BSA, 27 (51%) surpassed the normal range, while this number was 50 (94%) for absolute VO_2peak_. There were no significant differences in classification between indexing to VO_2VT_, VO_2COP_ and absolute VO_2peak_.

**Table 4:** Proportions with enlarged LVEDV indexed to BSA, VO_2peak_, VO_2VT_, and VO_2COP_ using both age- and sex-specific and overall reference values

	Athletes, *n* = 39		Patients with heart failure, *n* = 53	
Enlarged LVEDV indexed to	Overall	Age and sex	Overall	Age and sex
VO_2VT_	2 (5%)	1 (3%)	51 (96%)	50 (94%)
VO_2COP_	1 (3%)	1 (3%)	49 (92%)	47 (89%)
VO_2peak_	1 (3%)	2 (5%)	50 (94%)	51 (96%)
BSA	8 (21%)	5 (13%)	27 (51%)	22 (42%)

Mean (SD); *n* (%).

BSA, body surface area; LVEDV, left ventricular end-diastolic volume; VO_2VT_, oxygen uptake at the ventilatory threshold; VO_2COP_, oxygen uptake at the cardiorespiratory optimal point; VO_2peak_, peak oxygen uptake.

**Figure 3:**
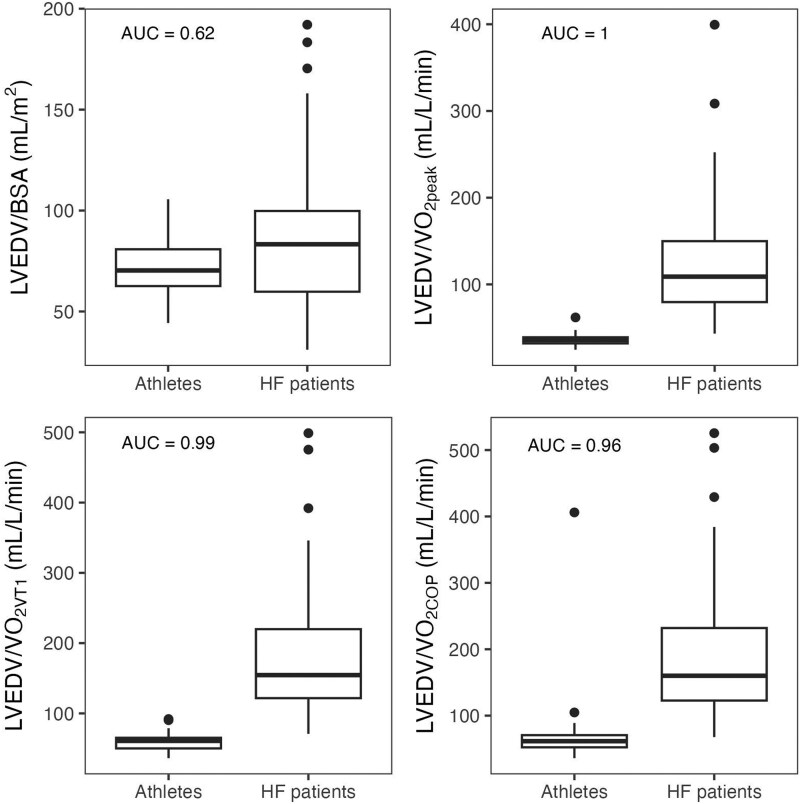
Box plots for LVEDV indexed to BSA, VO_2peak_, VO_2VT_, and VO_2COP_ for athletes and patients with HF.

Among the athletes, two (5%) individuals presented values outside the normal range when using upper limits for LVEDV indexed to VO_2VT_. This was a significant decrease (*P* = 0.042) compared to 8 (21%) classified as enlarged indexed to BSA. Only 1 athlete (3%) exceeded the limit of normalcy using VO_2COP_ and absolute VO_2peak_. Analyses comparing the AUC for LVEDV indexed for BSA, VO_2peak_, VO_2VT_, and VO_2COP_ showed AUC values of 0.62, 1.0, 0.99, and 0.96 for discrimination between athletes and patients with HF, respectively (*Figure 3*).

### Agreement between observers in VO_2VT_ assessment

Interobserver variability for 96 tests using Bland–Altman analyses revealed a bias between measurements of 0.06 with 95% limits of agreement of −0.55 to 0.66 L/min and a repeatability coefficient of 0.49 L/min (24%). The Pearson correlation coefficient was 0.85 (95% CI 0.78 to 0.89).

### Agreement in LVEDV measurements

For echocardiographic measurements of LVEDV the intra-operator repeatability coefficient was 27 mL (24%), the bias between measurements was −3.1 mL with 95% limits of agreement of −30.1 to 23.9 mL. The Pearson correlation coefficient was 0.93 (95% CI 0.87 to 0.96).

## Discussion

In a healthy sample from the general population, LVEDV was more strongly correlated with both VO_2VT_ and VO_2COP_, compared to BSA. Still, these submaximal measures being closely related to LVEDV, they showed a weaker correlation to LVEDV than VO_2peak_. Utilizing the submaximal measures for indexing LVEDV instead of BSA significantly increased the amount of patients with HF classified as enlarged, as well as the numbers of athletes classified as normal.

### Comparing VO_2peak_ and submaximal measurements

The VT is typically observed at a range of 45–74% of VO_2peak_ in healthy individuals,^15^ and normally at a higher percentage in endurance-trained individuals.^16^ The strong association between VO_2peak_ and LVEDV aligns with previous studies on the relationship between these two variables,^10,27–31^ and the findings are consistent with the described ability of cardiac structures to adapt to meet metabolic demands.^2^ Given the known, strong correlation between VO_2peak_ and VO_2VT_, the presented correlations between submaximal measures and LVEDV are not surprising. However, few studies have investigated the relationship between LV size and submaximal measures.

A study investigating the relationship between LV mass, LV end diastolic diameter and VT in male middle- and long-distance runners reported no significant correlation. However, only 60 participants were included, and the methods for data acquisition and analysis were not described in detail, limiting comparability with the present findings.^32^

A possible explanation why VO_2VT_ and VO_2COP_ do not demonstrate an equally strong association to LVEDV as VO_2peak_, may be the fact that absolute VO_2peak,_ as described by the Fick equation, is primarily limited by cardiac output (CO).^16,33^ CO is largely dependent on stroke volume (CO = heart rate × stroke volume, stroke volume = end-diastolic volume − end-systolic volume), which in turn is modulated by changes in LVEDV. Although cardiac structure and function are central determinants of the submaximal measures as well, peripheral factors such as metabolic adaptations in the skeletal muscles more heavily influence submaximal measures than maximal measures.^33,34^ Additionally, determination of VO_2VT_ is more prone to measurement variability than VO_2peak_, which may contribute to its weaker association with LVEDV.

When assessing the impact of age and sex, we found that LVEDV indexed to BSA was significantly influenced by both factors, underscoring the need for age- and sex-dependent reference values. VO_2peak_ exhibited less dependency on age and sex than BSA, whereas VO_2VT_ and VO_2COP_ showed even weaker associations, which may suggest greater robustness across demographic groups. Still, there were small differences compared to VO_2peak_, and VO_2peak_ explained a larger amount of variance even after the inclusion of age and sex for the submaximal measures. Nevertheless, prior studies have observed a significant age-related decline in VO_2VT_ for both sexes, yet at a slower rate than for VO_2peak_.^34–36^ This aligns with the findings in the present study, where the observed mean percentage of VO_2VT_ relative to VO_2peak_ increased with age. The influence of sex on VO_2COP_ has yet to be established, but large sex differences have generally not been reported.^21,31^ Due to a greater decline in VO_2_ relative to VE, COP (VE/VO_2_) typically increases with age. However, the magnitude of this age-related change has not yet been compared to declines in VO_2VT_ and VO_2peak_.^21,37^

### Classification of athletes and patients with heart failure

Although the results revealed a closer correlation between VO_2peak_ and LVEDV compared to the submaximal measures, there was no significant difference in these three indices’ ability to classify the hearts of the athletes as normal (*Table 4*). When using VO_2VT_, VO_2COP_, or VO_2peak_ 3–5% of them exceeded the upper limits, whereas 21% were classified as enlarged when indexed to BSA, which currently serves as the recommended indexing variable in both the general population and among athletes.^5,6^ This finding further highlights how LVEDV in athletes can exceed normal reference ranges when scaled to BSA,^8^ underscoring the need for alternative LVEDV indexing methods in athletic populations—particularly when considering that the population in the present study consisted of recreational endurance athletes rather than elite competitors.

A key finding of the present study was that indexing LVEDV to VO_2VT_ and VO_2COP_, significantly increased the proportion of patients with HF classified with LV enlargement, classifying 96% and 92% to enlarged, respectively, compared to 51% using BSA. Indexing with submaximal measures showed equivalent improvements as indexing to VO_2peak_, although indexing to submaximal measures did not perform better and should primarily be conceived as a fallback alternative if maximal exercise testing is not achievable. The findings demonstrate that individuals with HF exhibit disproportionately large LV volumes relative to their cardiorespiratory fitness, likely reflecting pathological adaptations, initially aimed at compensating for a reduced capacity to sustain adequate stroke volume.^38^ However, patients with HF are not able to increase their EF sufficiently, nor their heart rate during exercise to the levels of healthy exercisers, limiting stroke volume, CO and thus VO_2peak_, as well as submaximal measures of fitness.^39^

A noteworthy finding was that the mean VO_2VT_ value of the patients with HF corresponded to 70% of VO_2peak_, exceeding that of both the healthy and athletic groups. Similar trends have been reported when CPET variables have been assessed in comparable populations,^40^ and the observed percentage aligns with what has been described in previous studies.^20,41^ The relatively high proportion of VO_2VT_ to VO_2peak_ in the HF population may be explained by their reduced maximal aerobic capacity and inability to maintain exercise after the VT, causing VO_2VT_ to manifest at a higher relative exercise intensity. Furthermore, factors such as β-blocker therapy,^40^ along with greater challenges in accurately determining VO_2VT_ among patients with HF—potentially due to greater prevalence of ventilatory abnormalities^42^ and increased likelihood of submaximal test performance—may collectively contribute to this upward shift in VO_2VT_ relative to VO_2peak_.^16,43^ Supporting this, also VO_2COP_ has been shown to occur at a higher percentage of VO_2peak_ in patients with HF compared to healthy individuals.^37^

### Alignments and differences between VO_2VT_ and VO_2COP_

Although both VO_2COP_ and VO_2VT_ represent submaximal indices of cardiorespiratory fitness, they differ in their physiological underpinnings and estimation approaches. VO_2VT_ and VO_2COP_ are described to occur at a range from 45–74% to 30–50% of VO_2peak_, respectively.^15,21^ In the healthy general population, they were found to align closely with the VO_2COP_ value, corresponding to 95% of VO_2VT_. Moreover, no significant differences were found in the correlation coefficient and *R*^2^ between LVEDV and the two measures, and they demonstrated comparable performance in classifying individuals across the athletic population and patients with HF. One clear benefit of using VO_2COP_ lies in its methodological simplicity. This is particularly relevant in populations where data quality may vary, such as among patients with. Definition of VO_2VT_ requires some interpretation, whereas VO_2COP_ and COP can be algorithmically defined by a rolling average of VE/VO_2_ and are less dependent on visual estimation, thereby reducing measurement bias and inaccuracy. Previous studies have emphasized this strength, pointing to COP’s reproducibility and reduced dependence on observer input.^21,37^

### Clinical implications

A key advantage of VO_2VT_ and VO_2COP_ over VO_2peak_ is that they are less effort dependent, making them more feasible in non-athletic populations, considering that achieving maximal effort can be challenging and sometimes impractical.^36^ Furthermore, submaximal assessments are valuable in cases where maximal exertion is contraindicated due to health concerns, such as in certain arrhythmogenic cardiomyopathies.^17–20^ Although instances where maximal but not submaximal testing is medically contraindicated may be relatively uncommon, premature termination of CPET due to patient-related factors like low motivation or discomfort is a frequent challenge in clinical settings, highlighting the advantage of having the option to use submaximal data when only these are available. Still, our results from sensitivity analyses suggest that VO_2peak_ values probably can be used at similar or better accuracy than submaximal values. However, in instances where CPET is terminated at a clearly submaximal effort, submaximal measures of fitness may be preferred.

Conducting CPET is more resource-intensive than calculating BSA based on easily obtainable body mass and height, which may limit its use to specific clinical scenarios with uncertainty regarding pathological adaptations. However, CPET is performed in several clinical scenarios, and these data support an integrative interpretation of findings from echocardiography and CPET in relevant clinical scenarios. Still, some clinical scenarios demand multiparametric approaches combining CPET and exercise stress echocardiography.^44^

### Limitations

While our findings provide insights into a minimally explored topic, certain limitations should be acknowledged. The estimation of submaximal fitness measures from CPET performed until voluntary exhaustion in this study implies that caution should be made when generalizing the findings to using true submaximal tests for estimating VO_2VT_ and VO_2COP_. Non-univocal determination of the VT (using both the V-slope method and ventilatory equivalent method) may present a limitation, and estimation of VO_2VT_ was performed by manual interpretation with following observer variability. The Bland–Altman analysis on interobserver measurement agreement demonstrated an agreement in VO_2VT_ estimations slightly lower than that reported by experienced readers.^45^ However, it is also worth noting that BSA, despite its widespread use, was calculated using the Dubois & Dubois formula, which is empirically derived based on very limited data,^1^ and that both VO_2VT_ and VO_2COP_ clearly outperformed BSA. The use of AI-driven interpretation approaches could improve the accuracy of and ease VO_2VT_ estimations. The voluntary participation of subjects in the study introduces a risk of selection bias, potentially limiting the generalizability of the findings. Similarly, the transferability across different ethnicities is unknown, as the dataset is primarily based on a Caucasian population. Since CPET was performed on a treadmill, the results may not be directly applicable to other exercise modalities.

It is important to acknowledge that the analyses on the classification of patients with HF and athletes were based on a relatively small sample, and the patients with HF had a low EF of 34.7%, thus differentiating these groups might have been a low bar. The ability to detect subtle pathologies such as evolving cardiomyopathies is unknown, and our results should not be generalized to such settings without further study. Optimally, these methods should be compared for their ability to predict clinical outcomes, which should be followed up in further studies. Furthermore, only recreational athletes were included, and the potential impact of involving elite athletes is thus not known. At last, the low proportion of females in the athlete (26%) and HF (17%) group limits generalizability of the findings.

## Conclusions

This study demonstrates that using submaximal measures of VO_2_ for indexation does not improve differentiation between physiological and pathological LV remodelling compared to using peak values, although it performed better than using traditional BSA-based indexing. Submaximal exercise testing could be a useful alternative when testing to maximal exertion is impractical or contraindicated, although these findings should be replicated using exercise testing aborted prior to voluntary exhaustion. Further research in larger and more diverse populations is warranted to evaluate the applicability for differentiating subtle pathologies from physiological adaption.

## Data Availability

The data from HUNT used in this study are available on application to the HUNT Data Access Committee in accordance with the policy on data availability (further information and contact information: https://www.ntnu.edu/hunt/data).

## Lead author biography

 Julie Stendahl Spets and Victoria Elisa Joakimsdatter Sørgjerd are both in their final year as medical students at the Norwegian University of Science and Technology.
